# Health-SigQOLM is a versatile scale for measuring various aspects of health-related quality of life

**DOI:** 10.1186/s13104-024-06823-7

**Published:** 2024-06-13

**Authors:** Mohamad Adam Bujang, Wei Hong Lai, Selvasingam Ratnasingam, Xun Ting Tiong, Yoon Khee Hon, Eileen Pin Pin Yap, Yvonne Yih Huan Jee, Nurul Fatma Diyana Ahmad, Alex Ren Jye Kim, Masliyana Husin

**Affiliations:** 1 Sarawak General Hospital, Ministry of Health Malaysia, Kuching, Malaysia; 2 Institute for Clinical Research, Ministry of Health Malaysia, Shah Alam, Malaysia; 3 Clinical Research Centre, Sarawak General Hospital, Jalan Hospital, Level 5, 93586 Kuching, Sarawak Malaysia

**Keywords:** Health-related quality of life, Clinical evidence, SigQOLM

## Abstract

**Objective:**

The “Health” element is one of the elements in Significant Quality of Life Measure (SigQOLM) that measures quality of life and well-being of people. This study aims to evaluate the Health element (Health-SigQOLM) as a generic and dynamic scale to measure health-related quality of life (HRQOL) with a broader spectrum of coverage. This study used a secondary data that developed SigQOLM. Only the “Health” element with 33 items is used for analysis.

**Results:**

The construct of Health-SigQOLM has a minimum factor loading of 0.425 with excellent model fit. The health status among healthcare workers is significantly associated with the Health-SigQOLM (*p* < 0.001). The Health-SigQOLM score can clearly distinguish between healthy people and those who have been afflicted with some diseases but have never been hospitalized due to disease progression or other associated complications (*p* = 0.002). The Health-SigQOLM is a generic and dynamic tool for assessing various aspects of health-related quality of life.

**Supplementary Information:**

The online version contains supplementary material available at 10.1186/s13104-024-06823-7.

## Introduction

Health is one of the most important elements in human life. Health is a multidimensional concept that encompasses the state of both body and mind, which shall collectively reflect on the overall body and mind functionality, and this forms the basis for defining an individual’s health quality [1]. Ideally, health is an outcome measure for determining an overall state of well-being which consists of many spectrums or domains and a combination of all these spectra shall represent the overall requirement for a standard of health-related quality of life (HRQOL) [2]. Therefore, HRQOL can often be regarded as a standard outcome measure for assessing an individual’s health status which is collectively defined by the overall state of well-being in various domains commonly experienced by an individual or group of individuals [3–8].

Previous dynamic and generic scales have been developed to measure HRQOL [3, 6]. However, the concept of HRQOL can in fact be further expanded to incorporate more diverse perspectives. For example, although the WHOQOL-BREF has addressed dimensions of sleep quality and body image; however, it only includes one item for each of the two dimensions while there are various other ways to portray to what extent a person has been satisfied with his/her sleep quality and body image [6]. In another example, the MOS SF-36 does not measure body image and sleep quality [3].

The Significant Quality of Life Measure (SigQOLM), developed in 2023, comprises four elements, 18 domains, and 69 items. One of these elements is “Health” (Health-SigQOLM), which consists of nine domains measured through 33 items. Originally designed to holistically assess the quality of life (QOL) and well-being of individuals within a two-week period [9], SigQOLM primarily focuses on health-related quality of life (HRQOL), reflecting its significance as a determinant of overall QOL and well-being. However, previous publications have predominantly focused on item and framework development, lacking clinical evidence for the health element. Consequently, this study aims to validate the health element in SigQOLM (Health-SigQOLM) through criterion (known group comparison) and concurrent validity assessments (correlation among various domains in Health-SigQOLM).

## Methods

This study utilized secondary data from the development of SigQOLM, focusing solely on the “Health” element for analysis. In the primary data collection phase, only those study respondents who provided informed consent to participate were surveyed [9]. Ethical approval for this study was granted by the Medical Research and Ethics Committee (MREC), Malaysia. The ethical approval number for this study is NMRR ID-21-01979-XDL (IIR).

Health-SigQOLM comprises nine domains with 33 items [9]. Healthcare workers representing a diverse range of health conditions had previously participated in this study by providing responses to all items within the Health-SigQOLM questionnaire. The Health-SigQOLM items can be found in the Appendix 1. Additionally, the study recorded self-responses regarding current health conditions. These self-responses are deemed valid, as the respondents are healthcare workers familiar with various health conditions, and they undergo periodic health screenings at the hospital.

For this question, five different scenarios were created and respondents are required to choose only one category that best describes their current health status (i.e., each participant will be delegated to belong to any one of the following Category 1, Category 2, Category 3, Category 4, or Category 5). These responses were then categorized into three major categories such as ‘optimal health’ (Category 1), ‘mild infirmity’ (Category 2), and ‘poor health’ (Category 3, Category 4, and Category 5). These items that describe an individual’s current health status can be found in the Appendix 2.

### Statistical analyses

Although a previous study presented the construct and model fit for the “Health” element in SigQOLM, the detailed results were not provided [9]. Therefore, it is necessary to present the results pertaining to construct validity or factor solution in this paper. Exploratory Factor Analysis (EFA) was conducted to determine the total number of domains of ‘Health-SigQOLM’. The analysis utilized Maximum Likelihood Estimation (MLE) as the method of factor extraction, along with the Varimax Rotation method for simplifying and clarifying the data structure [9]. A factor solution was derived by adhering to Kaiser’s criterion, which stipulates that only factors with an Eigenvalue of > 1 would be retained. Confirmatory Factor Analysis (CFA) was then applied to evaluate the degree of model fit of Health-SigQOLM based on various absolute fit indicators, such as the Chi-squared goodness of fit (< 3.0), Root Mean Square Error Approximation (RMSEA < 0.08), and Standardized Root Mean Square Residual (SRMR < 0.08) [10].

An independent sample t-test and one-way analysis of variance test were also applied to determine the level of association between the Health-SigQOLM and the self-assessment measure of an individual’s current health status. Pearson’s correlation test was applied to determine the correlation between all domains and the standardized score of Health-SigQOLM to determine its concurrent validity. All the analyses were conducted by using SPSS (IBM Corp. Released 2011. IBM SPSS Statistics for Windows, Version 20.0. Armonk, NY: IBM Corp.) and R Core Team (R: A language and environment for statistical computing. R Foundation for Statistical Computing, Vienna, Austria. URL http://www.R-project.org/.).

## Results

A total of 406 respondents participated in the study and a majority of these respondents are female with 332 (81.8%) respondents. Overall, 285 (66.4%) participants were healthy without any existing comorbidities (Category 1), 97 (22.6%) participants with comorbidities (Category 2), and the remaining participants (11.0%) have existing comorbidities and are currently experiencing disease progression and complications (Category 3 – Category 5). The summary of results obtained by EFA reported the minimum factor loading to be 0.425 and the minimum value of Cronbach’s alpha for all the domains was found to be more than 0.749. Meanwhile, the summary of results obtained by CFA concluded that the Health-SigQOLM has displayed an excellent model fit (Table 1). The is also a relatively high correlation between all domains and the standardized score of Health-SigQOLM which can range from 0.512 to 0.755 (Table 2).

**Table 1:** Reliability, construct validity, and model fit of the Health-SigQOLM

	*n*	Factor loadings	Cronbach’s alpha	Chi-square test < 3.0	RMSEA < 0.08	SRMR < 0.08
Health-SigQOLM	33		0.961	2.201	0.054	0.075
Pain and discomfort	5	0.624–0.738	0.869			
Physical energy	4	0.425–0.843	0.890			
Emotional symptoms	3	0.643–0.683	0.866			
Independent	3	0.640–0.939	0.806			
Mobility	4	0.537–0.724	0.779			
Sleep quality	4	0.611–0.764	0.876			
Body image	4	0.694–0.854	0.914			
Eating regime	2	0.698–0.827	0.749			
Perception of health	4	0.653–0.830	0.914			

Note:

“n” refers to the total number of items retained in a factor solution based on Kaiser’s criterion for retaining only those factors with an Eigenvalue of more than one

**Table 2:** Concurrent validity between domains of Health-SigQOLM and the total standardized score of Health-SigQOLM

Domains	Coefficient
Pain	^0.699^
Physical strength	^0.755^
Psychological symptoms	^0.724^
Independent	^0.514^
Mobility	^0.512^
Sleep	^0.736^
Body image	^0.676^
Eating condition	^0.518^
Perception of future health	^0.633^

Noted: All results are statistically significant with *p* < 0.001

### Clinical evidence

The health status among healthcare workers is significantly associated with the overall score of Health-SigQOLM (*p* < 0.001) (Table 3). The results show that the Health-SigQOLM score can clearly distinguish between healthy (Category 1) people and those who have been afflicted with some diseases but have never been hospitalized due to disease progression or other associated complications (Category 2) (*p* = 0.002). Furthermore, pairwise multiple comparisons were conducted post hoc to test the difference between each pair of means (i.e. category 1 versus category 2, category 1 versus category 3, category 2 versus category 3) which detected that the scores among participants in Category 2 to be statistically significantly different from those scores obtained from those participants who have already experienced some disease progression and/or other associated complications (Categories 3, 4, and 5) (*p* < 0.001). As there were too few respondents who belonged to Categories 4 and 5, they were both included in Category 3.

**Table 3:** Association of Health-SigQOLM with three distinctly different categories of current health status (i.e. healthy, mild infirmity, poor health)

Based on the scenario of health condition	Mean (SD)	*p*-value
Category 1 (Healthy) Category 2 (Mild Infirmity) Category 3–5 (Poor Health)	82.4 (10.3)	< 0.001
	78.4 (10.8)	
	71.5 (10.8)	
One-Way ANOVA Post-hoc tests by pairwise multiple comparisons (LSD*)		
Category 1 vs. Category 2 Category 1 vs. Category 3–5 Category 2 vs. Category 3–5		0.002
		< 0.001
		< 0.001

Note:

Category 1: Healthy and without any pre-existing comorbidity(ies)

Category 2: With comorbidity (ies) but have never been hospitalized due to disease progression or complications

Category 3: With comorbidities (ies) and have already been hospitalized due to disease progression or complications

Category 4: With comorbidities (ies) and have already been hospitalized at least three times due to disease progression or complications

Category 5: Dependent on medicine and medical procedure(s) and/or medical equipment as a life support tool to maintain life (i.e. major surgery, renal dialysis, blood transfusion for thalassemia, heart transplant/stent placement, chemotherapy for cancer, etc.

*LSD: Least Significant Difference

## Discussion

Currently, there is a limited range of existing available HRQOL scales and most of these scales had already been developed more than 20 years ago [3–7]. It is now necessary to improvise the HRQOL scale by designing a better version that is designed to measure a broader spectrum of health concerns. Hence, the Health-SigQOLM shall serve as a more versatile HRQOL instrument for us to assess the HRQOL of both healthy and non-healthy individuals which shall then extend its use in an accurate assessment of HRQOL as part of the effort in the development of innovative intervention and treatment programs.

Since the assessment of HRQOL is dynamic in nature, the Health-SigQOLM is developed to measure HRQOL experienced by people during the immediately preceding two weeks. This scale consists of nine domains that cover important health-related factors that are originally obtained from many existing HRQOL scales but with a much broader scope of coverage [3, 6, 9]. The Health-SigQOLM has a high level of construct with excellent model fit. The high correlation between all the domains and the overall score of Health-SigQOLM also supports its concurrent validity. Apart from garnering the usual statistical evidence of its reliability and validity, this study also found the existence of a statistically significant association between the Health-SigQOLM and its domains with the current status of health conditions. All this cumulative evidence indicates that Health-SigQOLM is sufficiently sensitive to differentiate between the different categories of current health status. Therefore, the Health-SigQOLM has met most of the requirements expected of a scale property [11]. In conclusion, it is established that the Health-SigQOLM is a reliable and valid scale for assessing dynamic and generic Health-Related Quality of Life (HRQOL).

Unlike MOS SF-36, WHOQOL-BREF includes various other environmental factors such as freedom, safety, financial security, etc [6]. . . In this case, WHOQOL-BREF is also assessing a broader spectrum of QOL measures beyond the usual health matters. Even though it is an indisputable fact that such environmental factors are also important for an assessment of QOL; however, these factors do not directly provide a standardized measure of an individual’s health status. This explains why the WHOQOL-BREF is deemed preferable for use as an instrument for gauging an individual’s general aspects of QOL with a particular focus on HRQOL. Hence, it is recommended to exclude all the items from the environmental domain of WHOQOL-BREF if a researcher intends to use WHOQOL-BREF as an instrument to merely assess patients’ health-related outcomes for research studies involving the implementation of any intervention or medication.

Although WHOQOL-BREF has included many factors; however, a majority of these factors are represented by only one item such as eating, sleeping, body image, and future health. In our attempt to improvise on WHOQOL-BREF, the Health-SigQOLM include at least two items for representing each domain. This allows each domain to be measured fairly and constructively, and to minimize the likelihood of responder bias which can arise due to exerting a particular focus on any one facet of a domain only. For example, sleep quality is measured based on a list of various requirements such as satisfaction with sleep quality, whether or not having difficulty getting to sleep, the total number of hours available for sleeping, and whether or not feeling tired after waking up from sleep. Besides content validity, the construct of all the domains in Health-SigQOLM was also validated by various statistical tests, all of which would lead to an accrual of statistical evidence for its validity [9].

This study recommends future researchers continuous explore the possibility of enhancing the wide applicability of the Health-SigQOLM by translating it into various other languages and also applying it in real-life clinical practice and research. Clinicians and researchers can use the Health-SigQOLM as an outcome measure for determining the real impact of any interventional studies to determine the effectiveness of a particular medication or treatment. The policymakers and other key stakeholders can then use the Health-SigQOLM to obtain an accurate measure of the health-related quality of life of both normal healthy people as well as real patients in a bid to select a list of appropriate and optimal policy decisions for improving people’s quality of life.

As for conclusion, the Health-SigQOLM consists of nine domains with 33 items can be used to measure an individual’s generic and dynamic HRQOL over the course of the immediate past two weeks. The association between the overall score of Health-SigQOLM and its clinical evidence was found to be statistically significant, thereby demonstrating its clinical utility.

### Limitations

The study sample for this study only includes healthcare workers. An ideal sample to be obtained from patients with various health conditions. Therefore, future studies can explore the feasibility and versatility of using Health-SIGQOLM to accurately assess the health outcomes from both healthy individuals and real patients. Despite having such a limitation, the favourable results obtained from this study have nevertheless found that the Health-SigQOLM is able to discriminate status of health’s condition among the respondents.

### Electronic supplementary material

Below is the link to the electronic supplementary material.

**Supplementary Material 1**: **Appendix 1**: Questionnaire of Health Significant Quality of Life measures (Health-SigQOLM) and scoring mechanism for the Health-SigQOLM. **Appendix 2**: Additional question on measuring health status.

## Data Availability

The datasets used and/or analysed during the current study are available from the corresponding author on reasonable request.

## Abbreviations

EFA: Exploratory Factor Analysis
EQ-5D: EuroQol scale with 5 domains
CFA: Confirmatory Factor Analysis
HbA1c: Hemoglobin A1C
HRQOL: Health-related quality of life
LSD: Least Significant Difference
MOS SF-36: Medical Outcome Study 36-item short form survey
QOL: Quality of life
WHOQOLBREF: World Health Organization Quality of Life BREF
