# Supplementary material for: Health-SigQOLM is a versatile scale for measuring various aspects of health-related quality of life
Source: BMC Res Notes. 2024 Jun 13;17:162. doi: 10.1186/s13104-024-06823-7 (PMC11170794; doi:10.1186/s13104-024-06823-7)
Supplement: Supplementary file 1 — Supplementary Material 1: Appendix 1: Questionnaire of Health Significant Quality of Life measures (Health-SigQOLM) and scoring mechanism for the Health-SigQOLM. Appendix 2: Additional question on measuring health status. [file 13104_2024_6823_MOESM1_ESM.docx]

**Appendix 1**

**Questionnaire of Health Significant Quality of Life Measures (Health-SigQOLM)**

***Sosal selidik Pengukuran Kualiti Hidup Kesihatan yang Bermakna***

**Instruction:**

**The aim of this questionnaire is to measure how you feel about your quality of life related to health and non-health conditions for the past TWO weeks. You will be asked how frequently certain things happened in your life for the past TWO weeks. Please read the statements carefully and mark (x) for each item as the most appropriate option for you.**

***Arahan:***

***Soal selidik ini bertujuan untuk mengukur kualiti hidup seseorang daripada keadaan kesihatan dan bukan kesihatan untuk DUA minggu yang lepas. Anda akan ditanya setakat mana kekerapan sesuatu perkara itu berlaku dalam hidup anda dalam masa DUA minggu yang lepas. Sila baca setiap soalan dengan teliti dan tandakan (x) untuk setiap soalan bagi menggambarkan pilihan terbaik anda.***

| No. | Item / *Perkara* | Never  *Tidak pernah* | Seldom  *Jarang* | Sometimes  *Kadang-kadang* | Normally  *Biasanya* | Always  *Sentiasa* |
| --- | --- | --- | --- | --- | --- | --- |
|  | 1. **Physical pain or discomfort/ *Kesakitan atau ketidak selesaan fizikal*** |  |  |  |  |  |
| 1 | I feel discomfort at any part of my body  *Saya rasa tidak selesa di mana-mana bahagian badan saya* | ( ) | ( ) | ( ) | ( ) | ( ) |
| 2 | I have to take medications  *Saya perlu makan ubat* | ( ) | ( ) | ( ) | ( ) | ( ) |
| 3 | I have to see a doctor  *Saya perlu berjumpa doktor* | ( ) | ( ) | ( ) | ( ) | ( ) |
| 4 | I feel pain at any part of my body  *Saya rasa sakit di mana-mana bahagian badan saya* | ( ) | ( ) | ( ) | ( ) | ( ) |
| 5 | I feel unhealthy  *Saya rasa tidak sihat* | ( ) | ( ) | ( ) | ( ) | ( ) |
|  | 1. **Physical energy/ *Kekuatan fizikal*** |  |  |  |  |  |
| 6 | I am easily exhausted when I do my daily activities (e.g. walking, working, etc)  *Saya mudah keletihan apabila melakukan aktiviti harian (cth. berjalan, bekerja, dll)* | ( ) | ( ) | ( ) | ( ) | ( ) |
| 7 | I am unable to carry heavy items which I used to be able to do previously  *Saya tidak dapat memikul barang berat seperti yang saya biasa lakukan sebelum ini* | ( ) | ( ) | ( ) | ( ) | ( ) |
| 8 | I am unable to perform heavy task which I used to be able to do previously  *Saya tidak dapat melakukan kerja berat seperti yang biasa saya lakukan sebelum ini* | ( ) | ( ) | ( ) | ( ) | ( ) |
| 9 | I feel lack of physical energy  *Saya rasa kurang tenaga fizikal* | ( ) | ( ) | ( ) | ( ) | ( ) |
|  | **3.0 Emotional/ *Emosi*** |  |  |  |  |  |
| 10 | I feel depressed  *Saya berasa tertekan* | ( ) | ( ) | ( ) | ( ) | ( ) |
| 11 | I feel anxious  *Saya berasa cemas* | ( ) | ( ) | ( ) | ( ) | ( ) |
| 12 | I am not motivated to pursue any activities  *Saya tidak bersemangat untuk melakukan sebarang aktiviti* | ( ) | ( ) | ( ) | ( ) | ( ) |
|  | **4.0 Independence/ *Kemampuan untuk berdikari*** |  |  |  |  |  |
| 13 | I depend on others to move from one place to another  *Saya bergantung kepada orang lain untuk bergerak dari satu tempat ke satu tempat yang lain* | ( ) | ( ) | ( ) | ( ) | ( ) |
| 14 | I need assistance from others to help me in my daily activities  *Saya memerlukan bantuan daripada orang lain untuk membantu saya melakukan aktiviti-aktiviti harian* | ( ) | ( ) | ( ) | ( ) | ( ) |
| 15 | I am dependent on others to fulfil my self-care needs  *Saya bergantung kepada orang lain untuk memenuhi keperluan penjagaan diri saya* | ( ) | ( ) | ( ) | ( ) | ( ) |
|  | **5.0 Mobility / *Pergerakan*** |  |  |  |  |  |
| 16 | I need an equipment (eg: walking stick, wheelchair, etc) to help me mobilize  *Saya memerlukan sesuatu peralatan (cth: tongkat, kerusi roda, dll) untuk bergerak* | ( ) | ( ) | ( ) | ( ) | ( ) |
| 17 | My movements are slower than people of my age  *Pergerakan saya lebih lambat daripada orang yang seusia dengan saya* | ( ) | ( ) | ( ) | ( ) | ( ) |
| 18 | I have difficulty climbing stairs  *Saya menghadapi kesukaran untuk menaiki tangga* | ( ) | ( ) | ( ) | ( ) | ( ) |
| 19 | I feel pain when I move  *Saya rasa sakit apabila saya bergerak* | ( ) | ( ) | ( ) | ( ) | ( ) |
|  | **6.0 Sleep quality/ *Kualiti tidur*** |  |  |  |  |  |
| 20 | I am not satisfied with my sleep quality  *Saya tidak berpuas hati dengan kualiti tidur saya* | ( ) | ( ) | ( ) | ( ) | ( ) |
| 21 | I have difficulty falling asleep  *Saya mengalami kesukaran untuk tidur* | ( ) | ( ) | ( ) | ( ) | ( ) |
| 22 | I sleep less than 6 hours a night  *Saya tidur kurang dari 6 jam pada waktu malam* | ( ) | ( ) | ( ) | ( ) | ( ) |
| 23 | I feel tired after I wake up from sleep  *Saya berasa letih selepas bangun daripada tidur* | ( ) | ( ) | ( ) | ( ) | ( ) |
|  | **7.0 Eating condition/ *Kondisi pemakanan*** |  |  |  |  |  |
| 24 | I am careful with what I eat  *Saya berhati-hati dengan apa yang saya makan* | ( ) | ( ) | ( ) | ( ) | ( ) |
| 25 | I need to control my diet  *Saya perlu mengawal diet (pemakanan) saya* | ( ) | ( ) | ( ) | ( ) | ( ) |
|  | **8.0 Body image / *Imej tubuh badan*** |  |  |  |  |  |
| 26 | I am not satisfied with my body image  *Saya tidak berpuas hati dengan imej bentuk badan saya* | ( ) | ( ) | ( ) | ( ) | ( ) |
| 27 | I am not satisfied with my weight  *Saya tidak berpuas hati dengan berat saya* | ( ) | ( ) | ( ) | ( ) | ( ) |
| 28 | I wish to change my body image  *Saya berharap dapat menukar bentuk imej badan saya* | ( ) | ( ) | ( ) | ( ) | ( ) |
| 29 | I don’t like my present body appearance  *Saya tidak suka penampilan saya sekarang* | ( ) | ( ) | ( ) | ( ) | ( ) |
|  | **9.0 Perception of future condition regarding own health / *Persepsi masa hadapan terhadap kesihatan kendiri*** |  |  |  |  |  |
| 30 | I am worried that I will suffer poor health within 5 years  *Saya bimbang saya akan menghadapi kesihatan yang teruk dalam masa 5 tahun* | ( ) | ( ) | ( ) | ( ) | ( ) |
| 31 | I am worried that my lifespan is shorter than people of my age  *Saya bimbang jangka hayat kehidupan saya lebih pendek daripada orang seusia saya* | ( ) | ( ) | ( ) | ( ) | ( ) |
| 32 | I am worried that my health will not improve  *Saya bimbang kesihatan saya tidak akan bertambah baik* | ( ) | ( ) | ( ) | ( ) | ( ) |
| 33 | I have no solution to improve my health  *Saya tidak mempunyai penyelesaian untuk menambahbaik keadaan kesihatan saya* | ( ) | ( ) | ( ) | ( ) | ( ) |

A scoring mechanism for the Health-SigQOLM

| Domains | Items | Scoring | Standardized score (%) |
| --- | --- | --- | --- |
|  |  |  |  |
| Pain (n=5) | 1,2,3,4,5 | 0 to 20 | (Raw score/20) x 100 |
| Physical strength (n=4) | 6,7,8,9 | 0 to 16 | (Raw score/16) x 100 |
| Psychological symptoms (n=3) | 10,11,12 | 0 to 12 | (Raw score/12) x 100 |
| Independent (n=3) | 13,14,15 | 0 to 12 | (Raw score/12) x 100 |
| Mobility (n=4) | 16,17,18,19 | 0 to 16 | (Raw score/16) x 100 |
| Sleep quality (n=4) | 20,21,22,23 | 0 to 16 | (Raw score/16) x 100 |
| Eating condition (n=2) | 24,25 | 0 to 8 | (Raw score/8) x 100 |
| Body image (n=4) | 26,27,28,29 | 0 to 16 | (Raw score/16) x 100 |
| Perception on future health (n=4) | 30,31,32,33 | 0 to 16 | (Raw score/16) x 100 |
| The standardized score of Health-SigQOLM |  | 0 to 132 | (Raw score/132) x 100 |

**Appendix 2**

**Additional question on measuring health status**

Status of health (choose and mark ONLY one answer)

Status kesihatan (pilih dan tanda SATU jawapan sahaja)

( ) I am healthy (never been diagnosed with any medical condition except mild fever or headache, and I have never been hospitalised except for child delivery).

*Saya sihat (tidak pernah disahkan dengan mana-mana penyakit kecuali demam atau pening kepala, dan saya tidak pernah dimasukkan ke hospital kecuali untuk melahirkan anak)*

( ) I have been diagnosed with one or more than one diseases but never been hospitalized (except for child delivery).

*Saya telah disahkan menghidap satu atau lebih daripada satu penyakit tertentu tetapi tidak pernah dimasukkan ke dalam hospital (kecuali untuk melahirkan anak)*

( ) I have been diagnosed with one or more than one diseases and have been hospitalized due to disease progression or complications.

*Saya telah disahkan menghidap satu atau lebih daripada satu penyakit tertentu dan telah dimasukkan ke dalam hospital kerana penyakit berkenaan semakin parah atau disebabkan komplikasi penyakit*

( ) I have been diagnosed with one or more than one diseases and have been hospitalized more than 3 times due to disease progression or complications.

*Saya telah disahkan menghidap satu atau lebih daripada satu penyakit tertentu dan telah dimasukkan ke dalam hospital lebih daripada 3 kali kerana penyakit berkenaan semakin parah atau disebabkan komplikasi penyakit*

( ) I am dependent on medicine and/or medical procedure(s) and/or medical equipment to keep me alive (e.g. major surgery, dialysis for end stage renal disease, blood transfusion for thalassemia, heart transplant/stenting for heart problem, chemotherapy for cancer, etc.)

*Saya sedang bergantung kepada ubat dan/atau prosedur perubatan dan/atau peralatan perubatan untuk meneruskan kelangsungan hidup (cth: pembedahan besar, dialysis, pemindahan darah untuk thalasemia, pemindahan jantung, penggunaan sten untuk masalah jantung, kimoterapi untuk kanser dan/atau lain-lain.)*
